# Effect of metabolic disorders on reactive gliosis and glial scarring at the early subacute phase of stroke in a mouse model of diabetes and obesity

**DOI:** 10.1016/j.ibneur.2024.12.002

**Published:** 2024-12-04

**Authors:** Julien Clain, David Couret, Matthieu Bringart, Olivier Meilhac, Christian Lefebvre d’Hellencourt, Nicolas Diotel

**Affiliations:** a Université de la Réunion, INSERM, UMR 1188 Diabète Athérothrombose Thérapies Réunion Océan Indien (DéTROI), Saint-Pierre 97410, France; b CHU de La Réunion, Saint-Pierre 97410, France

**Keywords:** astrogliosis, CSPG, MCAO, db/db mice, extracellular matrix, glial scar, microgliosis, gliosis

## Abstract

It is well recognized that type II Diabetes (T2D) and overweight/obesity are established risk factors for stroke, worsening also their consequences. However, the underlying mechanisms by which these disorders aggravate outcomes are not yet clear limiting the therapeutic opportunities. To fill this gap, we characterized, for the first time, the effects of T2D and obesity on the brain repair mechanisms occurring 7 days after stroke, notably glial scarring. In the present study, by performing a 30-minute middle cerebral artery occlusion (MCAO) on db/db (obese diabetics mice) and db/+ (controls) mice, we demonstrated that obese and diabetic mice displayed larger lesions (i.e. increased infarct volume, ischemic core, apoptotic cell number) and worsened neurological outcomes compared to their control littermates. We then investigated the formation of the glial scar in control and db/db mice 7 days post-stroke. Our observations argue in favor of a stronger and more persistent activation of astrocytes and microglia in db/db mice. Furthermore, an increased deposition of extracellular matrix (ECM) was observed in db/db vs control mice (i.e. chondroitin sulfate proteoglycan and collagen type IV). Consequently, we demonstrated for the first time that the db/db status is associated with increased astrocytic and microglial activation 7 days after stroke and resulted in higher deposition of ECM within the damaged area. Interestingly, the injury-induced neurogenesis appeared stronger in db/db as shown by the labeling of migrating neuroblast. This increase appeared correlated to the larger size of lesion. It nevertheless raises the question of the functional integration of the new neurons in db/db mice given the observed dense ECM, known to be repulsive for neuronal migration. Carefully limiting glial scar formation after stroke represents a promising area of research for reducing neuronal loss and limiting disability in diabetic/obese patients.

## Introduction

Metabolic disorders encompass a variety of pathologies such as hypertension, diabetes and obesity (Rochlani et al., 2017, Fahed et al., 2022). The global prevalence of metabolic diseases has increased from several decades (Chew et al., 2023) to reach 10.5 % and 38 % of the world's population for type 2 diabetes (T2D) and obesity respectively (Khan et al., 2020). Importantly, T2D is closely associated with obesity, with almost 80–90 % of diabetics being overweight or obese (Grant et al., 2021). Both metabolic diseases are well known to be a primary risk factor for stroke and to worsen its consequences (Heuschmann et al., 2015, Maida et al., 2020, Horn et al., 2023).

A stroke occurs every three seconds worldwide, affecting 12.2 million people each year, resulting in 5 million deaths, and making it the second leading cause of death worldwide (Donkor, 2018, Feigin et al., 2021). The number of stroke victims almost doubled between 1990 and 2019 (Feigin et al., 2021, Stroke Experts Collaboration Group, 2022). Ischemic stroke is the most common type of stroke accounting for around 80 % of stroke. It corresponds to a permanent or temporary interruption of blood flow by a clot in a specific region of the brain (Kuriakose and Xiao, 2020). This induces a hypoxic environment in a part of the brain characterized by glucose, oxygen and nutrient deprivation. Such an environment favors neuroinflammation, oxidative stress and regional apoptosis, all of which promoting neuronal degeneration, subsequent disabilities and possibly death (Broughton et al., 2009, Chen et al., 2011, Mukandala et al., 2016, Jayaraj et al., 2019). Unfortunately, approved pharmacological therapy is limited to intravenous thrombolysis, within 4.5 hours of ischemic stroke, using injections of recombinant tissue plasminogen activator (rtPA) (Mosconi and Paciaroni, 2022, Patil et al., 2022). Another treatment option is mechanical thrombectomy, which can be performed within 6 hours of the onset of symptoms (Saver et al., 2015). After ischemic stroke, the resulting neurodegeneration induce reactive gliosis (Gao et al., 2013, Burda and Sofroniew, 2014, Shen et al., 2021). This process is characterized by the activation of astrocytes and microglia in the injured hemisphere, leading to their migration and proliferation around and/or within the damaged area (Streit et al., 1999, Pekny and Pekna, 2014, Pekny and Pekna, 2016). Reactive gliosis is also associated with typical morphological change, such as ameboid transition for microglia and hypertropia for astrocytes. The dense tangle of astrocytic and microglial cells forms a glial scar around the damaged area (Sofroniew, 2005, Sofroniew, 2009, Sofroniew, 2015, Wang et al., 2018), the physiological functions of such a scar remains controversial (Yang et al., 2020, Han et al., 2022, Perez-Gianmarco and Kukley, 2023). Nevertheless, recent therapies based on glial cell modulation after stroke appear to be a promising avenue of research to promote the brain's repair mechanism, thereby enhancing recovery from brain injury (Zhang et al., 2020, Perez-Gianmarco and Kukley, 2023).

As previously mentioned, the risk of stroke is higher in diabetics and obese people (Kivimäki et al., 2017, Mosenzon et al., 2023). In laboratory models of stroke such as the middle cerebral artery occlusion (MCAO), hyperglycemia solely is able to exacerbate brain tissue damage and neurological deficits after stroke (Couret et al., 2018, Fadini and Cosentino, 2018, Yang et al., 2021). Although the effects of obesity and/or diabetes on the consequences of stroke are well documented, the impact of such metabolic disorders on brain repair mechanisms and glial scarring are not well documented. We have recently shown that diabetic and obese mice (db/db) exhibit increased astrogliosis and microgliosis leading to an exacerbated glial scar compared to control littermates (db/+) 3 days after stroke (Clain et al., 2024). This time point corresponds to the acute phase of stroke when initial damage occurs and reactive gliosis is well initiated. However, there are no data on the impact of metabolic disturbances on the subacute phase of stroke (starting from 7 days post-ischemia). Subacute phase is a key period after ischemia when major improvements (brain plasticity and recovery) can occur. It is also a phase of health risks due to uncontrolled immune activation and inflammation.

The aim of this study is to better characterize the effects of metabolic disturbances in the early subacute phase of stroke (7 days after ischemia) to determine whether the reactive gliosis process and glial scarring are exacerbated, normalized, or returned to normal levels compared to normal metabolic conditions. To this end, we performed a 30-minute cerebral ischemia using the MCAO protocol in a model of diabetic and obese mice (db/db) versus controls (db/+). We then performed a panel of immunostaining experiments in order to characterize for the first time the impact of diabetes and obesity on glial scar formation and ECM deposition 7 days after stroke. Thus, we focused our analyses on the infarct area size, neurological outcomes, astrogliosis, microgliosis and also on extracellular matrix protein synthesis with a similar methodology to our study done at the acute phase of stroke (3 days post-ischemia, (Clain et al., 2024) to have a comparative approach and a better picture of the reactive gliosis and glial scaring processes. It is also a first attempt to better understand the effect of metabolic perturbation on injury induced neurogenesis.

## Materials and methods

### Animals and ethics

Male diabetic and obese db/db mice (6-week-old males, 25 g) and their db/+ littermates were purchased from Janvier Laboratories (Saint-Berthevin, France) and maintained under standard conditions of light (light/12 h dark cycle), humidity and temperature. Mice were fed a standard chow diet. All experiments were carried out on 8-week-old mice, in compliance with French and European guidelines for the use of animals in research, approved by the local ethics committee for animal experimentation (APAFIS #32634_2021052612229123).

### Measurement of metabolic parameters

Blood glucose concentration was measured following tail blood sampling using a standard glucometer (OneTouch®). For fasting blood glucose measurement, mice were denied access to food for a period of 6 hours. In addition, an oral glucose tolerance test was performed using a 30 % glucose solution after 6 h of fasting, followed by tail blood sampling 15, 30, 45, 60, 90 and 120 min after gavage. Body weight was measured before and after the MCAO procedure to avoid excessive weight loss after the ischemic procedure.

### Middle cerebral artery occlusion (MCAO) procedure

Thirty-minute cerebral ischemia was performed on 24 mice following a procedure already described (Couret et al., 2018). Briefly, mice were anesthetized with isoflurane and kept on a heating pad to avoid hypothermia. Next, a silicone rubber-coated 7–0 monofilament (702056PK5, Scholz Group, Doccol Corporation, Sharon, MA, USA) was inserted through the right common carotid artery at the bifurcation of the right middle cerebral artery (MCA) and the right anterior cerebral artery (ACA) (Couret et al., 2018). After removal of the monofilament, mice were returned to their cages for 7 days. This kinetic time point was chosen because it is a key post-ischemic period with possible major improvements in function and capacity, but it remains a period when immune activation and inflammation still occur. We refer it as post-subacute phase.

Soft food pellets and easy access to water were provided postoperatively. Finally, the mice were euthanized and fixed with intracardiac perfusion of PBS (Phosphate Buffered Saline) containing 4 % paraformaldehyde (PBS-PFA, pH 7.4).

### Neurological evaluation

Using the Bederson neurological deficit scale (Bederson et al., 1986), the neurological score of db/+ and db/db mice was assessed at 24 h, 48 h, 72 h and 7 days post-stroke. For this purpose, the score was developed as follows: "0" for no deficit, "1" for mild forelimb weakness, "2" for severe forelimb weakness and constant rotation on the deficit side when lifted by the tail, "3" for obligatory rotation, "4" for unconsciousness and "5" for death.

### Tissue preparation

Mice were rapidly dissected to remove the brains, which were then fixed overnight at 4°C. They were then cryoprotected in PBS containing 30 % sucrose for 48 h at 4°C, before being embedded in Tissue-TEK® OCT (KMA-0100–00A, Newtown, United Kindom) and frozen at −80°C. Coronal sections (12 µm thick) were prepared using a cryostat (Leica CM 15 20, Wetzlar, Germany).

### Immunohistofluorescence

After drying, brain sections were washed in PBT (PBS containing 0.2 % Triton X100) and non-specific binding was blocked in PBT containing 2 % BSA (bovine serum albumin) for 1 hours. Brain sections were finally incubated overnight at room temperature in 0.5 % BSA-PBT with the respective primary antibodies (Table 1): goat polyclonal anti-GFAP (1:1000, abcam, ab53554, RRID:AB_880202), goat polyclonal anti-Iba1 (1:250, abcam, ab5076, RRID: AB_2224402), rabbit polyclonal anti-collagen IV (1:500, abcam, ab19808, RRID:AB_445160), mouse monoclonal anti-chondroitin sulfate CS-56 (1:200, abcam, ab11570, RRID: AB_298176), rabbit monoclonal anti-tenascin C (Ten-C) (1:100, abcam, ab108930, RRID:AB_10865908), rabbit polyclonal anti-activated caspase 3 (Cas 3) (1:200, abcam, ab13847, RRID: AB_443014) to label astrocytes (GFAP), microglia (Iba1), collagen IV (Col-IV), chondroitin sulfate (GSPG), tenascin C (Ten-C) and activated caspase 3 (Cas 3) respectively.

**Table 1:** Primary antibodies.

**Antibodies**	**Host**	**Type**	**Reference**	**RRID**
**Caspase 3**	Rabbit	Polyclonal	ab13847	AB_443014
**Col-IV**	Rabbit	Polyclonal	ab19808	AB_445160
**CSPG**	Mouse	Monoclonal	ab11570	AB_298176
**GFAP**	Goat	Polyclonal	ab53554	AB_880202
Hemoglobin*	Rabbit	Polyclonal	ab191183	AB_2650996
**Iba1**	Goat	Polyclonal	ab5076	AB_2224402
**MAP2**	Mouse	Monoclonal	mab3418	AB_11212326
DCX*	Rabbit	Polyclonal	Ab18723	AB_732011
**Ten-C**	Rabbit	Monoclonal	ab108930	AB_10865908

Note that “* ” means that antigen retrieval was performed

The following day, after several washes with PBT, the slides were incubated with the respective secondary antibodies (Table 2), for 1h30 min at room temperature. Finally, the sections were washed with PBT and mounted with Ibidi mounting medium (REF: 50001, Ibidi, Gräfelfing, Germany). Nuclear counterstaining was performed using 4′,6′-diamidino-2-phenylindole (DAPI) (1:1000) for 2 hours at room temperature.

**Table 2:** Secondary antibodies.

**Antibodies**	**Reference**	**RRID**
**Donkey anti-goat Alexa Fluor 488**	ab150129	AB_2687506
**Donkey anti-mouse Alexa Fluor 488**	ab150105	AB_2732856
**Donkey anti-mouse Alexa Fluor 594**	ab150108	AB_2732073
**Donkey anti-rabbit Alexa Fluor 488**	ab150073	AB_2636877
**Donkey anti-rabbit Alexa Fluor 594**	ab150076	AB_2782993

Antibodies specificity was already characterized by the manufacturers and was also assessed by morphological analysis of the labeled cells and the absence of any staining with primary antibody omission.

### Microscopy and image analyses

Images were taken with an S60 nanozoomer (Hamamatsu). Contrast and brightness were adjusted in Adobe Photoshop CS7 similarly between db+ / and db/db mice. Quantification of immunolabeling was performed by two independent, blinded researchers using ImageJ software and were treated similarly between db/+ and db/db mice.

Our Immunostaining results represent quantification of positive cells or the positive surface area stained by antibodies on brain sections and cross-sectional regions. For each brain, at least three consecutive sections were analyzed: the positively stained area was quantified at the regions of interest using Image J. Briefly, a threshold was applied, resulting in binarization of the images. The stained area was provided via an Image J "analyze particles" plugin.

For caspase 3, cell counting was performed manually in regions of interest from 3 consecutive sections for each brain.

The proportion of ramified, intermediate and ameboid microglia stained with anti-Iba1 was assessed in three consecutive sections per brain, as described previously (Clain et al., 2024).

Each point on the graphs corresponds to the average count (number of cells or positive area) made in different sections of the same animal.

### Statistical analysis

Graph Pad Prism was used for statistical analyses. The comparisons between db/+ and db/db groups were performed using a statistical Student’s *t*-test. All data are expressed as mean + /- SEM, and n values correspond to the number of animals. A p-value < 0.05 was considered as statistically significant. * p < 0.05; * * p < 0.001; * ** p < 0.005 and * ** * p < 0.0001.

## Results

### Metabolic parameters of db/db mice

**Fig. 1:**
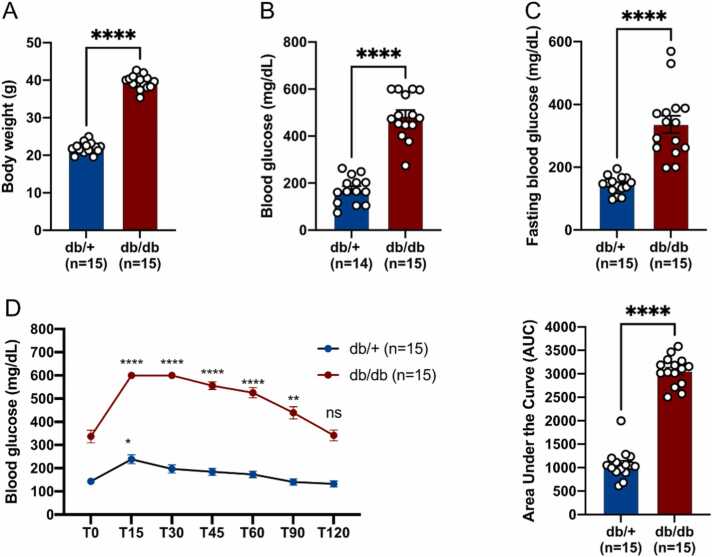
**Metabolic parameters of db/db and db/+ mice before MCAO, (A-C)** Body weight (A), blood glucose (B) and fasting blood glucose (C) measurements in db/db and db/+ mice. **(D)** Oral Glucose Tolerance Test (OGTT) demonstrating impaired glucose regulation in db/db mice compared with db/+ mice according to time (T0 to T120 minutes). Blood glucose levels peaked after 15 minutes in both groups. In each group, the blood glucose level measured is compared with the respective T0. It returned to basal levels after 30 minutes in control mice and after 120 minutes in the diabetic group, * p < 0.05, * ** * p < 0.001.

First, the metabolic disruption of the 8-week-old db/db mice was ascertained before proceeding to brain ischemia. As expected, body weight, blood glucose and fasting blood glucose levels were higher in db/db mice compared to their respective db/+ controls (Fig. 1 A-C). An oral glucose tolerance test (OGTT) using a 30 % glucose solution was performed. As observed, there is a peak of hyperglycemia 15 minutes after the glucose gavage in both groups. However, in control mice, there is a transient increase in blood glucose levels, which returned to baseline after 30 minutes. In the diabetic group, blood sugar levels were less well regulated, returning to normal only after 120 minutes, which is also confirmed by the higher area under the curve (AUC) (Fig. 1 D). Together, these results demonstrated that 8-week-old db/db mice exhibit metabolic dysfunctions.

### Disrupted metabolic parameters are associated with increased brain damage and limited recovery at distance of stroke (early subacute phase)

We next sought to characterize the impact of diabetes and obesity state on brain repair mechanisms by doing a 30 min MCAO in db/db and db/+ mice and maintaining them in optimal conditions for seven days to investigate brain damage, cell death, neurological score and reactive gliosis.

**Fig. 2:**
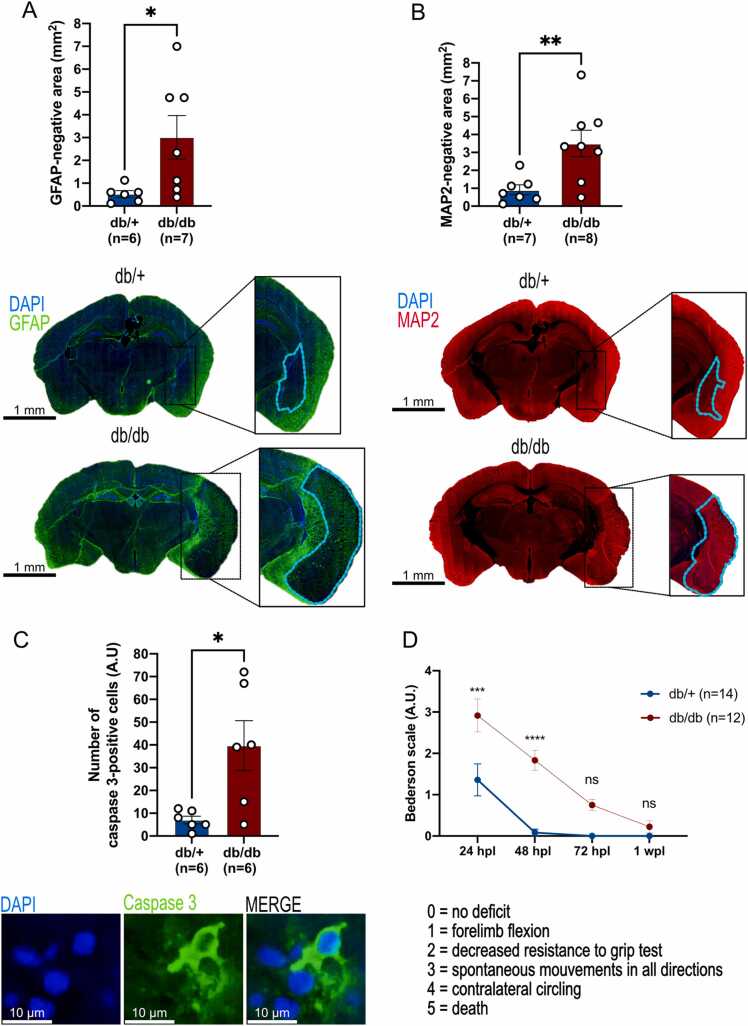
**Brain damage and cell death are aggravated in db/db mice after stroke, (A)** Representative images of GFAP staining (green) in db/+ and db/db mice showing the damaged area. Quantifications of GFAP-negative areas in the ipsilateral hemispheres (blue lines) allowing to determine the size of the ischemic area (blue demarcation lines). **(B)** Quantifications of MAP2-negative area documenting the size of the ischemic core in both mice groups (blue demarcation lines). Injured area and ischemic core are both larger in db/db mice compared to db/+ mice (graphs). **(C)** Number of activated caspase 3-positive cells and representative staining in the ischemic core of both groups, showing that db/db mice displayed higher number of apoptotic cells compared to db/+ mice. **(D)** Neurological score using the Bederson’s deficit scale demonstrating that the deficit was is significantly higher in db/db mice than in db/+ at 24 h and 48 h post lesion (hpl). * p < 0.05, * * p < 0.01, * ** p < 0.005, * ** * p < 0.001, scale bar: 10, 50 µm and 1 mm.

The size of the ischemic core was determined after MAP2 immunostaining that labels neurons. The MAP2-negative zone was quantified as a reflect of the ischemic region, as indicated previously (Arvola et al., 2021). In parallel, GFAP immunostaining was performed to label astrocytes. The GFAP-negative area surrounded by astrocytes corresponds to the total damaged area, and was quantified as described previously (Shimada et al., 2012). Together, the results showed that the ischemic core and global damage area were larger in db/db mice (Fig. 2A and B). Similarly, we quantified the number of apoptotic cells in the injured hemisphere in both groups. We observed a significant higher number of caspase 3-positive cells in the ipsilateral hemisphere of diabetic vs control mice (Fig. 2C).

Finally, the Bederson scale was used to determine the functional recovery of mice after stroke. As shown in Fig. 2D, db/db mice had a significant greater neurological deficit following stroke at 24 and 48-hours post-lesion (hpl), remaining higher at 72 hpl and returning to the basal neurological score of db/+ at 7 days post-stroke.

In conclusion, the poor metabolic conditions of db/db mice are linked to exacerbated brain lesions, cell death and neurological outcomes, illustrating the deleterious impact of hyperglycemia/obesity on infarct size and neurological recovery of db/db mice.

### Metabolic disorders and reactive gliosis at the early subacute phase of stroke

After central nervous system injury, reactive gliosis occurs leading to the formation of the glial scar (Huang et al., 2014, Yang et al., 2020). During this process, astrocytes and microglia are activated (i.e. proliferation, migration and phenotypical change) around and within the damaged area. The main cellular change occurring during astrogliosis includes hypertrophy and overexpression of glial fibrillary acidic protein (GFAP) (Sofroniew, 2005, Sofroniew, 2009, Sofroniew, 2015, Poulot-Becq-Giraudon et al., 2022). For microgliosis, it involves an ameboid transition and the up-regulation of the Iba1 protein (Bachiller et al., 2018, Lier et al., 2021). These physiological responses to brain injuries result in the formation of the glial scar (Gao et al., 2013, Karve et al., 2016, Zheng et al., 2023).

**Fig. 3:**
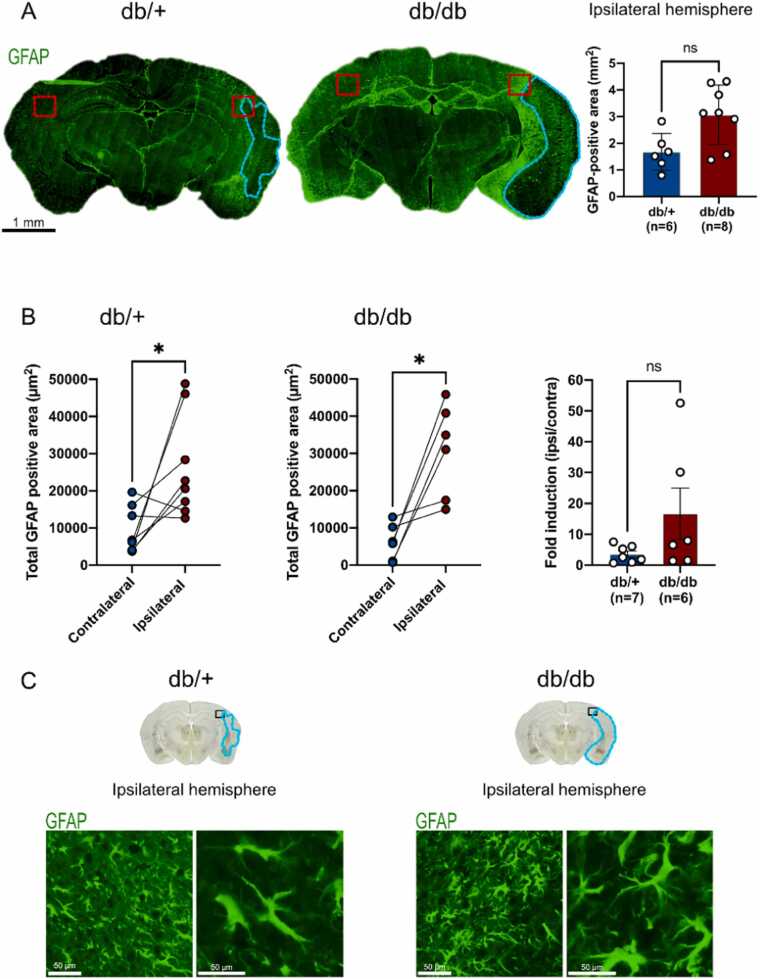
**Astrogliosis is increased in db/db mice 7 days after MCAO, (A)** Representative images of GFAP staining (green) in db/+ and db/db mice. Quantifications of GFAP-positive areas in the injured hemisphere indicated a trend towards increased GFAP reactivity in db/db compared to db/+ . **(B)** Perilesional quantification (red squares) of GFAP-positive area is significantly higher in the ipsilateral hemisphere compared to the contralateral hemisphere in db/+ and db/db mice. In contrast, GFAP fold induction (ipsilateral/contralateral) in diabetic mice did not reach statistical significance. **(C)** In the ipsilateral hemispheres, hypertrophy of astrocytic extensions and bodies was observed in diabetic and control mice at the perilesional level (black squares). Note that neuroanatomical regions had to be adapted in both groups because of the differences in location and size of the injured area. The brain schematic shows the location of the regions of interest studied (black squares) in the contralateral and ipsilateral parts of the db/+ and db/db, with the damaged area shown in blue. * p < 0.05, ns: non-significant.

**Fig. 4:**
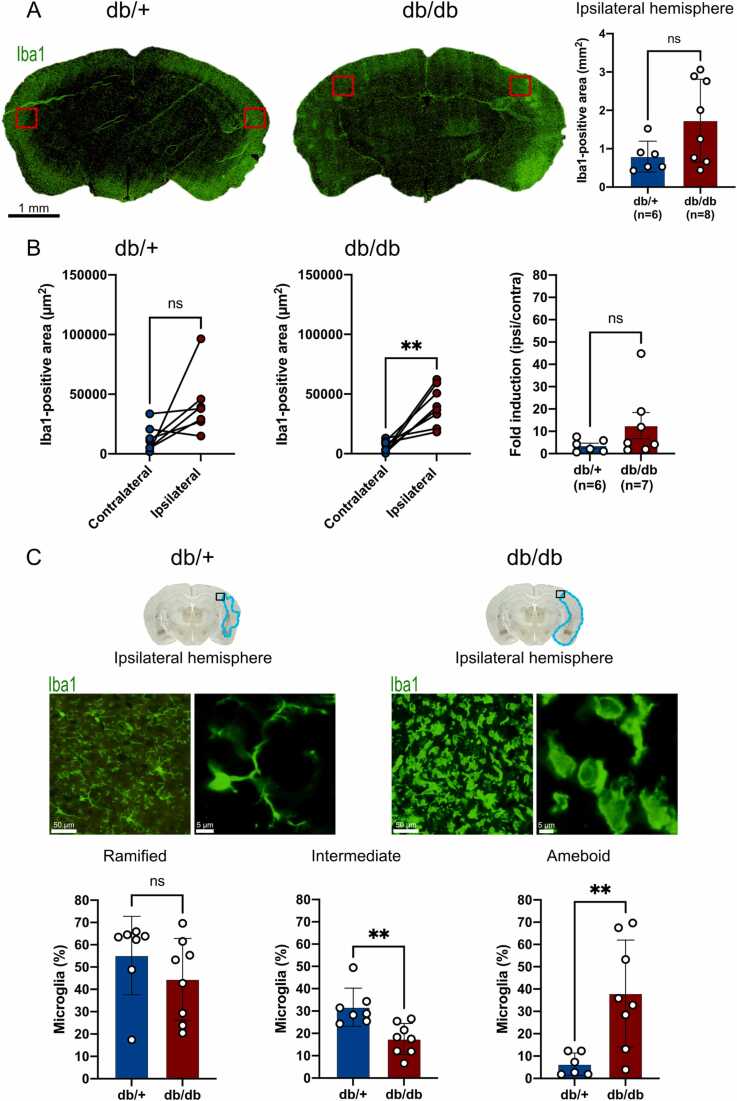
**Microgliosis is impaired in db/db mice after MCAO, (A)** Representative images of Iba1 staining (green) in db/+ and db/db mice illustrating microgliosis. Quantifications of Iba1-positive areas in the injured hemisphere. **(B)** Perilesional quantification (red squares) of Iba1-positive area is significantly higher in the ipsilateral hemisphere of diabetic mice, while this is not the case for db/+ mice. Iba1 fold induction (ipsilateral / contralateral) tended to be increased in db/db mice. **(C)** Representative pictures showing hypertrophic microglia (Iba1 in green) in the ipsilateral hemisphere in both groups. Ameboid microglia with a circular morphology can be observed in diabetic mice and stellate microglia in control mice. The quantification of ramified ("quiescent"), intermediate and ameboid ("activated/phagocytic") microglia demonstrates the sustained microglia activation in db/db mice, characterized by a notable reduction in intermediate microglia and a corresponding increase in ameboid ones. Note that neuroanatomical regions had to be adapted in both groups because of the differences in location and size of the injured area. The brain schematic shows the location of the regions of interest studied (black squares) in the contralateral and ipsilateral parts of the db/+ and db/db, with the damaged area shown in blue. * * p < 0.01, ns: non-significant, scale bar: 5, 50 µm and 1 mm.

We investigated the effect of metabolic disorders on astrogliosis and microgliosis in the subacute phase of stroke, a key period after stroke when neuroinflammation remains critical (Fig. 3, Fig. 4). As shown, the GFAP-positive area is larger in the ipsilateral than in the contralateral hemisphere in both groups (Fig. 3A and B) at the border of the damaged region. No significant differences in astrocytic activation were observed between db/db and db/+ mice. (Fig. 3B). On the basis of these results, we investigated the reactivity of astrocytes at higher magnification at the border of the damaged area. It seems that astrocytes display hypertrophic morphology in the injured hemisphere of diabetic mice, close to the area of damage, compared with their control counterparts (Fig. 3C). Together these data tend to show that db/db maintain more astrogliosis than db/+ for in the subacute phase of stroke.

Taken together, these results show that the metabolic conditions in the db/db mice correlate with increased astrogliosis in the injured hemisphere. At the vicinity of the damage area, the GFAP fold induction is higher in db/db than in db/+ . Obviously, a stronger density of GFAP staining was observed in the whole ipsilateral hemisphere of db/db mice, due to the size of the infarct.

We next investigated microgliosis which is a key process during brain damage and healing. By performing Iba1 immunostaining on brain sections, a strong and persistent presence of Iba1-positive cells was observed in the perilesional and damaged regions (Fig. 4A). The recruitment of microglial cells in the ischemic hemisphere compared to the contralateral one was significantly higher in the ipsilateral hemisphere of db/db mice. In contrast, in db/+ mice, no differences could be observed suggesting a resolution of the inflammatory process. This indicates persistent gliosis, and demonstrates the maintenance of a stroke-induced neuroinflammatory state in db/db in the 7 days after ischemia. Interestingly, the ratio of activation (ipsilateral/contralateral) did not reach statistical significance in db/db mice compared to db/+ probably in link to some sample heterogeneity (Fig. 4B).

In agreement with this increased number of microglia in the ipsilateral hemisphere of db/db mice (Fig. 4 A and B), the assessment of microglia reactivity at higher magnification clearly demonstrated their ameboid morphology in diabetic/obese mice (Fig. 4C). The morphology of microglia is a direct reflection of their activation status. In a homeostatic state, microglia display a ramified morphology, whereas an activated/phagocytic phenotype microglia exhibits a shift towards an ameboid morphology (Woodburn et al., 2021; Lull and Block, 2010; Davis et al., 2017; Dong et al., 2021). The ratio of ramified, intermediate and ameboid microglia at the damage site was quantified, thereby providing clear evidence of a differential activation of microglia between db/+ and db/db mice at the subacute phase of stroke. While db/+ mice exhibit a low percentage of ameboid microglia (<7 %), db/db mice display a persistent high number of ameboid cells (>35 %), arguing in favor a persistent neuroinflammatory state.

Overall, these results demonstrated that the metabolic conditions of db/db mice are closely linked with the persistent increased presence and activation of microglia in the subacute phase of stroke (7 days post-ischemia) in the damaged hemisphere compared to db/+ mice. These data are reinforced by morphological analyses and quantification confirming that the db/db condition seems to be associated with microglia activation in the subacute phase of stroke in diabetic mice.

### Metabolic disorders maintain ECM accumulation at the subacute phase of stroke

In the days following brain injury, activated cells secrete molecules and proteins into the extracellular matrix (ECM) (Frik et al., 2018, Kjell and Götz, 2020). This process allows to reduce the spread of lesions (Wang et al., 2018), but it also reduces brain plasticity by limiting axonal growth and brain repair (Silver and Miller, 2004, Fitch and Silver, 2008). We consequently investigated the expression of the main proteins involved in glial scarring notably Collagen IV (Col-IV), Chondroitin sulfate of proteoglycans (CSPG) and Tenascin C (TEN-C).

**Fig. 5:**
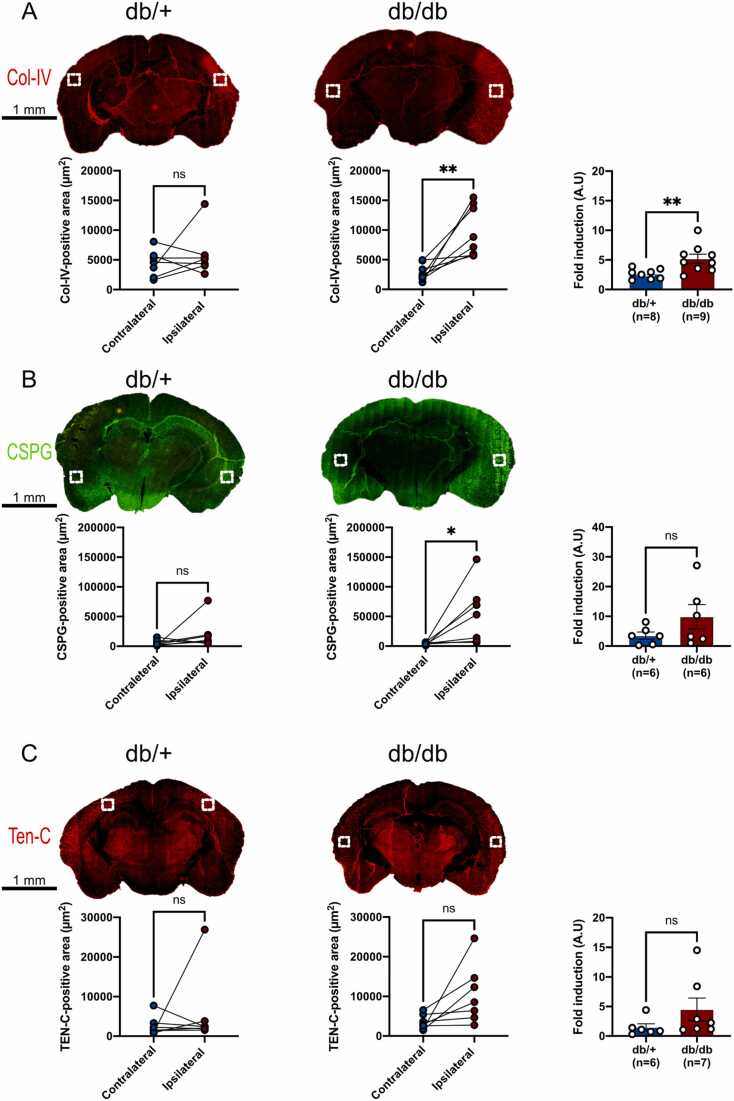
**Synthesis of type IV collagen and CSPG is enhanced in db/db mice after MCAO, (A)** Representative images of type IV collagen staining (Col-IV in red) in both groups. Quantifications of Col-IV-positive areas was performed in the ischemic core and in the corresponding area in the contralateral hemisphere (regions indicated by the white squares). Col-IV-positive area is significantly higher in the ipsilateral hemisphere in diabetic mice compared to the contralateral one 7 days post-stroke. Col-IV-fold induction (ipsilateral / contralateral) is higher in the db/db group compared to db/+ mice. **(B)** Representative images of chondroitin sulfate of proteoglycan staining (green) in both groups. Quantifications of CSPG-positive areas was performed in the ischemic core and in the corresponding area in the contralateral hemisphere (regions indicated by the white squares). In db/db mice, CSPG-positive area is significantly higher in the ipsilateral hemisphere of diabetic mice. CSPG fold induction (ipsilateral / contralateral) is unchanged between both groups. **(C)** Representative images of tenascin C staining (red) in both groups. Quantifications of TEN-C-positive areas was performed in the ischemic core and in the corresponding area in the contralateral hemisphere (regions indicated by the white squares). Ten-C fold induction (ipsilateral / contralateral) was comparable in both groups. * p < 0.05, * * p < 0.01, ns: non-significant, scale bar: 1 mm.

Quantification of Col-IV-positive area was performed in the ischemic core in both groups (Fig. 5A). Col-IV-positive area was not significantly increased in the ischemic hemisphere compared to the contralateral one in db/+ (Fig. 5A). In contrast, db/db mice had a significantly larger Col-IV-positive area in the injured hemisphere compared to the contralateral one (Fig. 5A). This was confirmed by Col-IV ratio quantification that was also higher in diabetic/obese mice (Fig. 5A).

Quantification of the CSPG-positive area confirms the same trend for each group of mice (Fig. 5B). Indeed, CSPG were not significantly upregulated in the ischemic hemisphere of db/+ 7 days after stroke, but were significantly increased in db/db mice (Fig. 5B). The CSPG ratio was also higher in diabetic/obese mice (Fig. 5B).

Concerning TEN-C, no significant differences were observed within each group of mice, even if the TEN-C ratio (ipsilateral / contralateral) tends to be increased in db/db compared to db/+ (Fig. 5C).

### Metabolic disorders exacerbate BBB leakage and hemorrhagic transformation, at distance of stroke (early subacute phase)

**Fig. 6:**
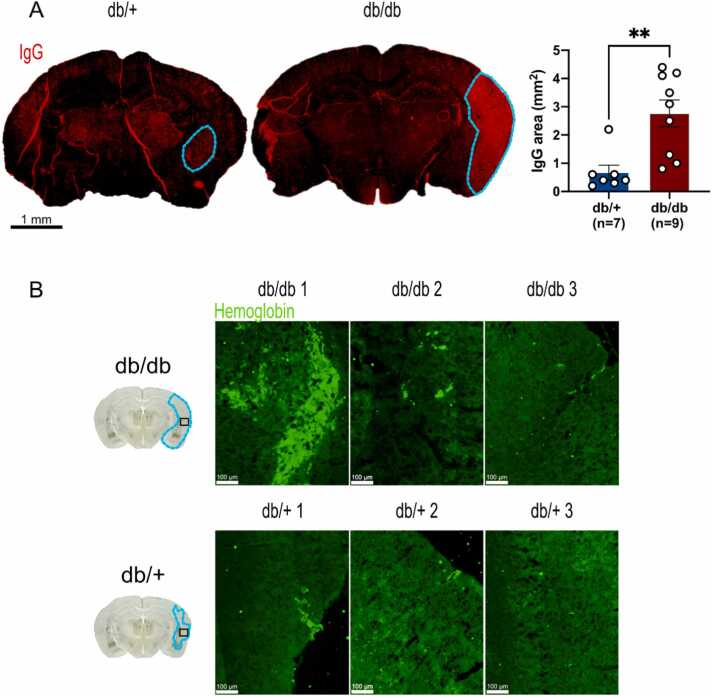
**Increased BBB breakdown and hemorrhagic transformation 7 days post-stroke in db/db mice, (A)** Representative pictures of IgG-positive area in db/+ and db/db mice. Note that the blue lines correspond to the diffusion of IgG in the brain following stroke, attesting of BBB breakdown. Quantification indicates that the positive zone for IgG is almost 4 times higher in db/db mice than in control mice. **(B)** Representative images of the hemoglobin-positive zone in the ischemic core in both groups. It seems that hemorrhagic transformation is more important in db/db mice. The brain schematic shows the location of the regions of interest studied (black squares) in the contralateral and ipsilateral parts of the db/+ and db/db, with the damaged area shown in blue. * * p < 0.01, scale bar: 1 mm.

In the event of brain damage, the blood-brain barrier (BBB) is compromised (Okada et al., 2020). This breakdown can be reflected by the diffusion of immunoglobulin G (IgG) within the brain parenchyma (Manwani et al., 2014). Quantification of IgG-positive area revealed a larger diffusion of IgG in db/db mice than in db/+ mice (Fig. 6A). Interestingly, BBB disruption is often associated with hemorrhagic transformation (HT), a major complication of reperfusion (Arba et al., 2020). We observed a greater hemoglobin staining in diabetic/obese mice, with nevertheless an important heterogeneity (Fig. 6B).

Overall, these results indicate that the metabolic state of db/db mice is associated with BBB leakage and hemorrhagic transformation 7 days after of stroke.

### Metabolic disorders and reactive neurogenesis

**Fig. 7:**
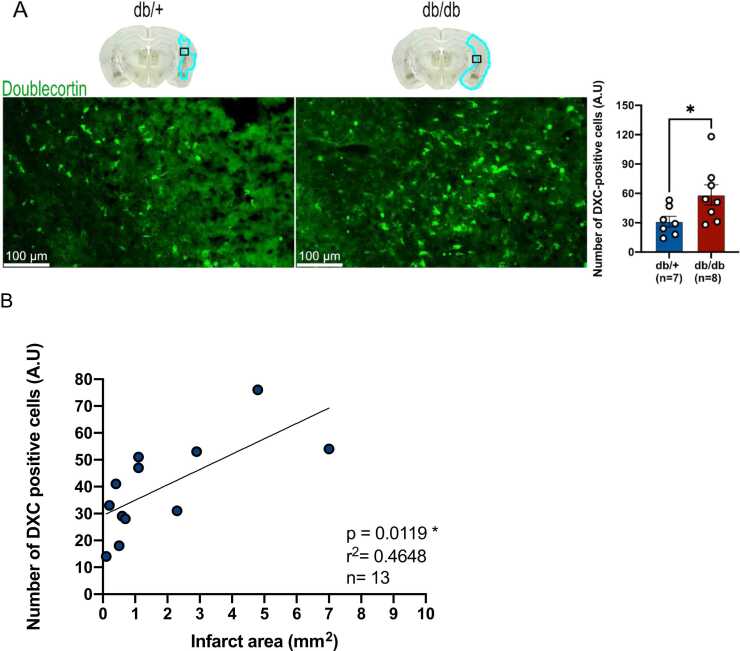
**Increased reparative neurogenesis in the injured area 7 days after stroke in db/db mice, (A)** Representative pictures of DCX-positive area in db/+ and db/db mice. Quantification indicated that the number of DCX-positive cells in the perilesional zone was twice as high in db/db mice as in db/+ mice. **(B)** Graphical correlation showing that the number of DCX-positive cells increases with the infarct volume (db/+ and db/db mice). The brain schematic shows the location of the regions of interest studied (black squares) in the contralateral and ipsilateral parts of the db/+ and db/db, with the damaged area shown in blue. * p < 0.05, scale bar: 100 µm.

In response to brain damage, injury-induced neurogenesis (regenerative neurogenesis) is a key component of brain plasticity and regeneration (Arzate and Covarrubias, 2020, Diotel et al., 2020). We assessed the expression of doublecortin (DCX), a well-known marker of adult neurogenesis. This protein is expressed by neural precursor cells (neuroblasts) and immature neurons (Couillard-Despres et al., 2005). The results indicated a significant higher number of DCX-positive cells in the perilesional region of db/db mice compared to db/+ mice (Fig. 7A). Given that injury-induced neurogenesis is normally correlated to the size of the brain damage, we assume that this process was increased in db/db mice due to the larger size of the lesion. As indicated, the number of DCX-positive cells is positively correlated with the infarct volume (Fig. 7B).

## Discussion

In this study, we sought to describe for the first time the impact of overweight-induced diabetes on (1) reactive gliosis and glial scarring, (2) extracellular matrix deposition and persistence and (3) regenerative neurogenesis at the early subacute phase of stroke (7 days after ischemia). As expected, we showed that under poor metabolic condition, infarct volume and ischemic core were significantly larger associated with worsen neurological functions and exacerbated BBB leakage as well as hemorrhagic transformation (HT). We also observed that diabetic mice exhibited persistent astro- and microgliosis in the damaged hemisphere, linked to increased synthesis of extracellular matrix proteins. As a reflect of regenerative neurogenesis, the number of DCX-positive neuroblasts migrating to the damage area was higher in the perilesional regions of db/db mice compared to db/+ ones, and appeared correlated to the size of cerebral infarct. Based on our data, we suggest that the disrupted metabolic condition displayed by db/db mice worsens outcomes after stroke by accentuating the glial scarring mechanism and by delaying the resolution of neuroinflammation.

### Brain damage, BBB leakage and neurological outcomes at the subacute phase of stroke

The effects of metabolic disorders (chronic hyperglycemia/overweight) on brain damage and neurological outcomes in the post-acute phase of stroke (>24 h) are poorly studied, most studies focusing on the acute phase of stroke first 24 hours to 48/72 hours (Johnston et al., 2019). Consequently, therapeutic treatments of diabetic and/or obese patients after brain injury remain limited.

In this study, we characterized the links between poor metabolic condition (obesity/hyperglycemia) and cell death as well as neurological outcomes 7 days post-ischemia (early subacute phase of stroke). This time was chosen because it is considered to be a critical period of initiation for brain recovery and plasticity. Unsurprisingly, db/db mice have larger infarct volume and ischemic core, due in part to increased apoptotic processes as revealed by stronger activated caspase 3 staining. These results are in agreement with our previous ones obtained at the acute phase of stroke (3 days post-ischemia) (Clain et al., 2024). Such deleterious effects of metabolic disruption on cell survival following stroke was also reported in different models such as high-fat diet models at 1 (Grisotto et al., 2021) and 7 days post-injury (Tulsulkar et al., 2016), and also in db/db mice at 14 days post-injury (Jiang et al., 2018). Consequently, the metabolic condition of db/db mice is associated with exacerbated cell death after stroke. However, it is interesting to note that at 3 days post-ischemia, corresponding to the acute phase of stroke, the damaged area (GFAP-negative) was 2 and 6 mm^2^ in db/+ and db/db mice, respectively (Clain et al., 2024), and decreased to about 0.5 and 3 mm^2^ in db/+ and db/db, respectively, in the early subacute phase of stroke (7 days post-ischemia, this study). So far, it appears that in db/+ , the area of damage is reduced by about 4, while in diabetic mice it is reduced by only 2. This suggests that diabetic and obese mice have a worse recovery phase at the histological level, and may need further investigation for confirmation.

In agreement with the increased brain damage observed, a greater neurological deficit was found in db/db mice 24 and 48 hours after MCAO as previously shown (Chen et al., 2016, Kurita et al., 2020). Three days post-stroke, this neurological score remained slightly higher than in db/+ but did not reach a significant level. Interestingly, 7 days after ischemia, both groups of mice showed similar neurological deficits. These results contrast with previous studies in which db/db mice showed persistent and significant neurological deficits at these same times (Tureyen et al., 2011, Akamatsu et al., 2015). The number of mice studied, their heterogeneous response to injury, the different occlusion durations (30 minutes (our experiment), 45 minutes (Tureyen et al., 2011), 90 minutes (Akamatsu et al., 2015)) and the different neurological tests applied may explain such discrepancies.

Together, these data raise the question of the respective role of type II diabetes (hyperglycemia) and overweight/obesity on brain damage since db/db mice are known to be obese and diabetic (Heydemann, 2016, Reilly et al., 2022). By way of an answer, induction of acute hyperglycemia have already demonstrated a worsening of oedema, hemorrhagic transformation and a larger infarct volume in non-diabetic and non-obese mice 1 and 2 days after the stroke (Khan et al., 2016, Couret et al., 2018, Chen et al., 2022). Hyperglycemia alone clearly appears to be an independent factor aggravating the neurological consequences of stroke.

Also, we noticed that the breakdown of the blood-brain barrier (BBB) was exacerbated 7 days after stroke in db/db mice compared to db/+ . This result perfectly fits with those of Jiang and colleagues (2020) reporting a stronger IgG extravasation in the acute phase of stroke at 2 days after ischemia (Jiang et al., 2020). In agreement, hemoglobin staining was in general higher in the ischemic hemisphere of db/db mice, reflecting a greater hemorrhagic transformation (HT). Interestingly, a strong heterogeneity was observed for HT in db/db group. Exacerbated hemorrhagic transformation has already been showed in other models of metabolic disruptions such as in HFD at 24 h post-stroke (Taïlé et al., 2022). Our results coincide with the well-characterized time course of BBB degradation, which peaks at 3 days and persists for up to 14 days post-stroke (Kang et al., 2020). We therefore assumed that the metabolic condition of db/db mice aggravated the breakdown of the BBB 7 days after stroke. This could imply that BBB disruption could persist longer in case of metabolic dysfunction.

Our observations in mice are in agreement with clinical investigations evidencing larger infarct volume (Watila et al., 2014), increased BBB leakage and hemorrhagic transformation (Kerenyi et al., 2006, for the Neurological Emergencies Treatment Trials Network and the SHINE Trial Investigators, 2019, Spronk et al., 2021) in diabetic patient after ischemic stroke. Such an impact of hyperglycemia worsens unfailingly neurological deficits of diabetic patient after stroke (Megherbi et al., 2003). Despite the fact that obesity is well-known to be heavily associated with diabetes (Klein et al., 2022, Ruze et al., 2023), its impact after brain injury remains controversial (on behalf of the FAST-MAG Investigators and Coordinators, 2021, Quiñones-Ossa et al., 2021, Kim and Choi, 2023, Lee et al., 2023). In this context, diabetes appears to play a key role in stroke pathology and identifying how it exacerbates the consequences of stroke could help to reverse its negative effects and thus promote better recovery in stroke patients.

## Persistent increase in astrogliosis and microgliosis at the early subacute phase of stroke (7 days post-ischemia) in db/db mice

Cerebral injuries are known to drive the activation of astrocytes (astrogliosis) and microglia (microgliosis) (Karve et al., 2016, Donat et al., 2017, Michinaga and Koyama, 2021), resulting in a dense network around the injured area called the glial scar (Sofroniew, 2015, Moulson et al., 2021). However, the links between chronic hyperglycemia/overweight and this process remains poorly documented.

To this aim, we investigated the glial scar at 7 days post-stroke. In our experimental conditions, we analyzed the area covered by GFAP-positive astrocytes in the injured hemisphere at the close vicinity of the ischemic core. The rate of astrocyte activation, was consistently increased (almost 2 times higher) in db/db mice compared to db/+ 7 days after stroke. In addition, astrocytes from db/db mice appear to exhibit larger processes in the injured hemisphere than db/+ mice. Interestingly, a decrease in *GFAP* mRNA expression 1 day after ischemia was shown in db/db mice and was associated with a delayed activation of astrocyte (Kumari et al., 2007). Compared to our previous work at the acute phase of stroke (3 days post-ischemia) (Clain et al., 2024), our results on GFAP staining at the subacute phase (7 days) demonstrate that reactive gliosis is maintained in db/db mice. Together, these data argue in favour of a stronger and persistent activation of astrocytes in db/db mice in the post-acute phase of stroke probably in link with a higher brain damage size and delayed activation of astrocytes.

Regarding microglial activation, we also observed higher Iba1-positive microglia area in the ipsilateral hemisphere of db/db mice compared with the contralateral hemisphere in the subacute phase of stroke. The ratio of microglia activation (ipsi/contralateral) was twice as high in db/db mice as in db/+ mice. More importantly, microglia in db/db exhibit an ameboid phenotype in the peri-infarct region compared to those in db/+ one. The decreased proportion of intermediate microglia and, more notably, the increased proportion of ameboid microglia observed in the brains of db/db mice compared to their controls indicate the presence of a profound and persistent microglial activation following stroke in db/db mice. This may be attributed to either delayed activation or delayed resolution. In our previous work at 3 days post-ischemia (acute phase of stroke), the Iba1 staining was significantly higher in both ischemic regions of db/+ and db/db mice (Clain et al., 2024). Here, at the subacute phase (7 days post-ischemia), we observed that the Iba1 staining remained significantly increased in the ipsilateral hemisphere of db/db mice while it is back to normal in db/+ mice. Taken together, these data confirm the persistent activation of these neuroinflammatory cells in disrupted metabolic conditions at distance of stroke. Interestingly, it was previously shown that microglial activation was greater in the injured hemisphere of db/+ mice than in db/db at 1 day post-MCAO (Kumari et al., 2007). Nevertheless, our results are consistent with the kinetic of microglial activation peaking at 7 days post stroke (Li et al., 2013, Anttila et al., 2017, Buscemi et al., 2019). Together, these data show that microglial activation was accentuated in db/db mice at distance from stroke, suggesting that the persistent neuroinflammation could participate in the poorer recovery observed in diabetic patients.

Overall, our study emphasizes the effect of chronic hyperglycaemia/overweight in exacerbating astro- and microgliosis in the post-acute phase of stroke (7 days post-ischemia) a key phase of brain plasticity. To our knowledge, no study has yet described glial scarring in diabetic patients following stroke. In normoglycemic conditions, a post-mortem study showed an increase in the reactivity of astrocytes and microglia in the peri-infarct cortical region forming a glial scar after stroke (Huang et al., 2014). Given that hyperglycemia worsens neurological deficits and exacerbates glial scar formation after stroke, we hypothesized that reducing the thickness of the glial scarring may improve neurological outcomes of diabetic/obese patients as observed previously in non-diabetic rats (Bachiller et al., 2018).

## Effects of disrupted metabolism on ECM deposition at the subacute phase of stroke

After brain damage, reactive astrocytes and microglia among others, participate in the deposition of ECM around and in the ischemic core (Dias et al., 2021, Li et al., 2023). These cells mainly synthesize collagens, tenascins and chondroitin sulphate of proteoglycans (CSPG) (Moeendarbary et al., 2017, De Castro Brás and Frangogiannis, 2020, Li et al., 2023). In our work, we observed a greater surface covered by Col-IV, CSPG and Ten-C in the injured hemisphere of db/db mice compared to the contralateral ones. These increased expressions were statistically significant for CSPG and Ten-C in db/db mice. In our previous work, at the acute phase of stroke (3 days post-ischemia), Col-IV, CSPG and Ten-C were increased. These data demonstrate that the fibrotic scar is maintained in db/db mice at distance of stroke. Our data are also consistent with previous ones showing increased Col-IV synthesis in peri-infarct regions one day after the stroke (Härtig et al., 2017) and even at 14 days after stroke (Williamson et al., 2021) in non-obese and non-hyperglycemic mice. The results also indicate that hyperglycemia/overweight exacerbates Col-IV synthesis 7 days after stroke. There is a real lack of data showing the effects of metabolic disorders on CSPG and Ten-C in the post-acute phase of stroke. Further characterization of both molecules, Ten-C and CSPG, at this time window are needed regarding their respective implication in brain damage, neurological outcomes (Okada et al., 2020, Chelluboina et al., 2022) axonal budding and brain recovery (Luo et al., 2022).

Comparing these observations with clinical studies is somehow very complicated, given the difficulty of studying the temporal evolution of the extracellular matrix post mortem (Baeten and Akassoglou, 2011, Li et al., 2023), the different localization and duration of stroke in humans and the effects of diabetes and obesity on such processes. Among the few clinical studies available, it has been shown that dead patient following ischemic stroke displayed abundant CSPG deposition (Huang et al., 2014) and increased Col-IV expression in the infarct region (Michalski et al., 2020). We can therefore hypothesize that hyperglycemia in diabetic patients promotes the formation of a dense tangle composed mainly of astrocytes and microglia (thicker glial scar), which increases ECM deposition and consequently contributes to the worsening of post-stroke consequences. Reversing the effects of hyperglycemia on ECM could improve functional recovery of diabetic patients. Although no treatment exists that targets this extracellular matrix, there is growing interest in developing therapies that modulate ECM to improve recovery after stroke (Dzyubenko et al., 2018a, Dzyubenko et al., 2018b). In addition, it is conceivable that a reduction in the cell density and the thickness of the glial scar after a stroke could allow a better access to migrating neuroblasts in the oligemic zone and encourage hitherto limited regeneration.

## Effects of disrupted metabolism on regenerative neurogenesis at the early subacute phase of stroke

After brain injury, regenerative neurogenesis occurs in order to replace dead neurons (Xiong et al., 2010, Rahman et al., 2021, Uspenskaya et al., 2022). In a final series of experiments, we decided to investigate the effect of the diabetic environment of db/db mice on the regenerative neurogenesis. Hence, we observed a higher number of neuroblasts in the damaged region of db/db mice compared to db/+ one. Such a result has already been reported in non-obese and non-hyperglycemic mice where the migration of neuroblasts peaked at 7 days post-stroke in the injured hemisphere (Palma-Tortosa et al., 2017). However, it is well-known that injury-induced neurogenesis is positively correlated to the size of stroke. Regarding the increased size of stroke in db/db mice, it suggests that the increased regenerative neurogenesis observed in db/db mice could be due to the larger infarct area. We confirmed that, whatever the metabolic conditions, the number of DCX-positive cells significatively correlates with infarct volume. However, it remains to determine how such metabolic conditions, and its impact on the glial scar formation could affect the survival, differentiation, and functional integration of such new neurons within the damage area.

In humans, studies also show a higher number of neuroblasts in the damaged hemisphere of a patient who died following an ischemic stroke (Martí-Fàbregas et al., 2010). However, the impact of metabolic condition on regenerative neurogenesis is not known in humans and to our knowledge, there is no study investigating the post-mortem regenerative neurogenesis in a diabetic patient who has died following an ischemic stroke. Nevertheless, our results pave the way for new therapies favoring the mechanism of regenerative neurogenesis 7 days after stroke to improve recovery in diabetic patients.

## Conclusion

In summary, our results demonstrate for the first time that the poor metabolic condition related to chronic hyperglycemia and overweight of db/db mice is associated with exacerbated brain damage and neurological score, correlated with stronger astrogliosis, microgliosis and ECM deposition at the early subacute phase of stroke (7 days post-ischemia). These data showed that compared to the acute phase of stroke (3 days post-ischemia), brain recovery appears to be limited (brain size) and is associated with persistent astrogliosis and microgliosis in db/db mice at the subacute phase. Such persistent microgliosis may be associated with persistent inflammation that could impair regenerative processes that were initiated. Although the respective role of hyperglycemia and obesity on brain damage remains to be further investigated, the increased astrogliosis, microgliosis and glial scarring process observed in db/db mice could be the consequences of the increased infarct size. Limiting and/or reducing astrogliosis, microgliosis and ECM deposition in diabetic and obese patients could reverse theses adverse effects on glial scar formation and could represent a promising line of research for improving the recovery of diabetic patients after a stroke episode, which has so far been very limited.

## Fundings

This work was funded by European Regional Development Funds (RE0022527) ZEBRATOX (10.13039/100016077EU-Région Réunion-French State national counterpart), Grant/Award Number: RE0022527

## Animals and Ethics

All experiments were carried out on 8-week-old mice, in compliance with French and European guidelines for the use of animals in research, approved by the local ethics committee for animal experimentation (APAFIS #32634_2021052612229123).

## CRediT authorship contribution statement

**Christian Lefebvre d’Hellencourt:** Writing – review & editing, Writing – original draft, Validation, Supervision, Project administration, Methodology, Investigation, Conceptualization. **Nicolas Diotel:** Writing – review & editing, Writing – original draft, Visualization, Validation, Supervision, Project administration, Methodology, Investigation, Funding acquisition, Formal analysis, Conceptualization. **Julien Clain:** Writing – review & editing, Writing – original draft, Visualization, Validation, Methodology, Investigation, Formal analysis. **David Couret:** Writing – review & editing, Writing – original draft, Visualization, Supervision, Project administration, Methodology, Conceptualization. **Matthieu Bringart:** Writing – review & editing, Writing – original draft, Methodology, Investigation. **Olivier Meilhac:** Writing – review & editing, Writing – original draft, Validation, Supervision, Project administration, Methodology, Investigation, Conceptualization.

## Declaration of Generative AI and AI-assisted technologies in the writing process

During the preparation of this work Deepl was used to improve the English in some parts of the work. After using this tool/service, the author(s) reviewed and edited the content as needed and take(s) full responsibility for the content of the publication.

## Declaration of Competing Interest

The authors declare no conflict of interests.
